# Wake-Up Receiver-Based Routing for Clustered Multihop Wireless Sensor Networks

**DOI:** 10.3390/s22093254

**Published:** 2022-04-23

**Authors:** Maximilian Weber, Ghofrane Fersi, Robert Fromm, Faouzi Derbel

**Affiliations:** 1 Smart Diagnostic and Online Monitoring, Leipzig University of Applied Sciences, Wächterstrasse 13, 04107 Leipzig, Germany; robert.fromm@htwk-leipzig.de (R.F.); faouzi.derbel@htwk-leipzig.de (F.D.); 2 Research Laboratory on Development and Control of Distributed Applications (ReDCAD), National Engineering School of Sfax, University of Sfax, Sfax 3038, Tunisia; ghofrane.fersi@redcad.org

**Keywords:** WSN, wake-up based sensors, clusters, multicasting, IoT, routing, latency-minimisation, energy-efficiency

## Abstract

The Wireless Sensor Network (WSN) is one of the most promising solutions for the supervision of multiple phenomena and for the digitisation of the Internet of Things (IoT). The Wake-up Receiver (WuRx) is one of the most trivial and effective solutions for energy-constrained networks. This technology allows energy-autonomous on-demand communication for continuous monitoring instead of the conventional radio. The routing process is one of the most energy and time-consuming processes in WSNs. It is, hence, crucial to conceive an energy-efficient routing process. In this paper, we propose a novel Wake-up Receiver-based routing protocol called Clustered WuRx based on Multicast wake-up (CWM), which ensures energy optimisation and time-efficiency at the same time for indoor scenarios. In our proposed approach, the network is divided into clusters. Each Fog Node maintains the routes from each node in its cluster to it. When a sink requires information from a given node, it’s corresponding Fog Node uses a multicast wake-up mechanism to wake up the intended node and all the intermediate nodes that will be used in the routing process simultaneously. Measurement results demonstrate that our proposed approach exhibits higher energy efficiency and has drastic performance improvements in the delivery delay compared with other routing protocols.

## 1. Introduction

The Internet of Things (IoT) is developing towards a significant and extensive technology. The vision of a global infrastructure of connected physical objects interacting anywhere and anytime is still one of the emerging concepts in the IT world. Due to their robust design and self-organising networking without the need for extensive infrastructure, Wireless Sensor Networks (WSNs) are the backbone of IoT. They are increasingly used in different applications, e.g., for monitoring and data transmission in areas such as structural and environmental monitoring [1,2]. Further, healthcare, as well as smart home applications, such as real-time indoor air quality monitoring [3] and on-demand indoor localisation [4,5], are realised through this technology in a cost-effective and low-maintenance way. WSN is also becoming increasingly important in indoor scenarios where accurate and reliable information about people’s activities and behaviour is collected and disseminated to support assisted living in home environments [6,7]. The provision of wireless sensors for use in the sense of IoT is accompanied by various technical challenges [8]. Energy consumption is the most important challenge since the lifetime of each sensor is tightly related to its energy-critical battery. Thus, new ways must be found to increase the energy-efficiency of sensors.

One of the major drawbacks of recent wireless sensors is the power consumption of Radio Frequency (RF) transceivers with approximately 10–30 mA for routing processes or radio transmissions. In order to allow an energy autarkic operation, a duty-cycled approach with fixed phases of transmitting, receiving, and sleeping are generally introduced [9,10]. This leads to the increase of system latency. This latency is not acceptable, especially in time-restricted or quasi real-time applications with reaction times in the range of some milliseconds to some seconds [11]. Researchers, such as in [12,13], are promoting power management to improve the battery life of individual nodes and extend the lifetime of the entire network.

A very promising solution to face these problems and challenges is the use of a novel, energy-autonomous, on-demand communication hardware. In combination with the application-specific main sensors (temperature, fire, etc.), the so-called Wake-up Receiver (WuRx) forms the enhanced sensor node [14]. In order to preserve energy, the sensor node can be set to the sleeping state until a signal is received by the WuRx to wake up the node. This strategy, when applied in the routing process, is energy efficient but has drawbacks when it is applied in time-critical applications. Effectively, such solutions require an additional time to wake up each intermediate node sequentially when it is needed in the routing process [15,16].

To overcome these limits, this paper proposes an energy-efficient and delay minimising routing strategy using asymmetric data transmission with multicasting wake-ups in a heterogeneous and clustered WSN. Our proposed approach is made up of two layers:

- The sensors layer: made up of sensors equipped with batteries, and in addition, each sensor node is equipped with a WuRx.
- Fog computing layer: made up of Fog Nodes. These nodes are much more powerful than sensor nodes and are placed physically near the sensor nodes to assist them and help them in their complex tasks [17,18]. Each Fog Node is responsible for a sensor cluster. It controls the data packets transmitted in the network between the different network structures (sink and cluster nodes). It maintains in its routing table all the source routes from each node in its cluster to it.

Based on the frequent interactions, the sink and Fog Node are main powered. At the time that a packet is to be exchanged, the sink transmits a Request Packet (REQ) to the Fog Node, which sends a Wake-up Packet (WuPt) to start Data Packet (DataPt) transmission. To improve the communication in time and energy aspects, we have integrated a multicast wake-up, which allows a simultaneous wake-up of a specific group of nodes. Therefore, the Fog Node can use a high data transmission power for directly reaching the destination node as well as the nodes acting as relays.

We implemented the proposed method in a real scenario using the parameters of the WuRx developed at the Leipzig University of Applied Sciences. Evaluations show that our proposed approach has drastically optimised the energy consumption and has reduced the communication delay. The rest of this paper is organised as follows. Section 2 describes the background. Section 3 presents the related work. Section 4 explains our proposed approach and Section 4 presents the measurement evaluation. Finally, Section 5 concludes this paper and points out future work.

## 2. Background

Since our proposed approach is based on WuRx sensors and source routing, we give in this section a general overview of these two concepts.

### 2.1. Wake-Up Receiver-Based Sensors

The WuRx is used for permanent monitoring of the radio channel in order to wake up the main radio for processing information during event-driven communication. The radio chip in a wireless node consumes most of the energy in comparison to the other components of the node (i.e., sensors, microcontrollers (MCU)), which leads to an unavoidable battery drain. Since the continual activity of the radio is often not necessary, switching to sleep mode can dramatically save energy. Initially, the main radio is off, and the WuRx is active in low power listening mode waiting for an incoming WuPt to wake up the whole sensor node. At this mode, the WuRx consumes only a few microamperes. This is in contrast to the power consumption of the transceiver, which is used as the main radio. Its consumption of tens of milliampere in the receiving mode represents a significant increase in power consumption compared to the WuRx. Thus, the use of the WuRx provides optimal conservation of the battery-powered sensor node energy reserves for years.

**Figure 1:**
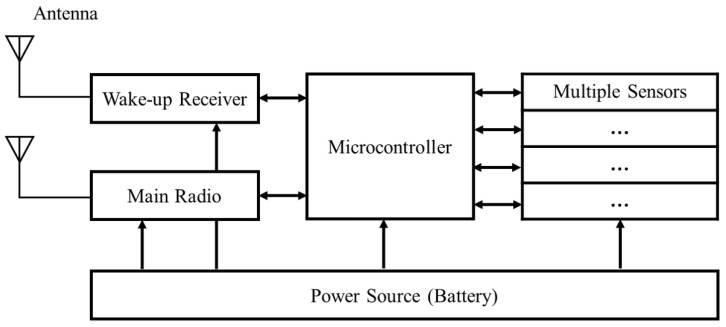
A wireless sensor node equipped with a WuRx, according to [19].

A sensor node is typically built out of antennas, WuRx, main radio, sensors, energy source, and microcontroller, as shown in Figure 1. The WuRx, which is an additional RF reception part, has its own antenna in order to receive only WuPt. The WuRx is configured by the microcontroller. The main receiver is used for the communication of data packages when the sensor node is in active mode. During standby, the WuRx is typically the only active component in the circuit. The WuRx analyses the incoming WuPt in order to validate the address. In the case of address matching of WuPt and WuRx, the WuRx sends an interrupt signal to the microcontroller to wake up.

### 2.2. Source Routing

Source routing is a technique that allows routing data packets to a specific destination. This routing strategy removes the decision making from the routers to individual nodes to learn about their neighbours and then choose the best possible paths when routing network traffic from one node to another. This source path contains all the addresses of the intermediate nodes responsible for forwarding the packet to the destination [20]. When a transmitter decides to communicate with a destination node, it checks its route cache to see if information about that destination is available. If the route cache does not contain such information for that destination, the transmitter initiates a route discovery process by sending a route request. If the route discovery is successful, the transmitter receives a route response packet with a number of intermediate nodes through which it can reach the destinations. Nodes can respond to requests even if they are not the destination to reduce delays. It is also possible for intermediate nodes to forward packets, to listen to the routes by examining the packets and finding out which routes lead to reliable destinations [21].

There are two possible source routing modifications that a node can make to a data packet as it goes onto the network: strict or loose. Strict source definitions specify the exact path a packet should take from one router to the next before the packet even leaves the source. Loose source routing, however, gives the packet some specific router points and lets the other routers decide along the way. This is useful, for example, when sending a packet through the routers of a local network to a gateway router and then to a specific address [22].

## 3. Related Work

The authors in [23] proposed two flooding protocols FLOODWUP and GREEN-WUP. FLOOD-WUP uses different broadcast addresses for opportunistic routing to forward messages to receivers that are not in range of the source node. Avoiding retransmission, each node changes its wake-up address upon reception of a data packet. To implement a further reliability mechanism, a unique sequence number is assigned to each broadcast packet. However, it may happen that a node loses the correct sequence, and additional control packages are required to reorganise the communication procedure. In GREEN-WUP, the researchers have added a parameter to the address of the sensor nodes in order to determine which sensor nodes should be used as relays. The address contains information about the level of harvested energy, based on which the sensor node with the highest level is selected. Unlike FLOOD-WUP, the sink goes into sleep mode after sending the WuPt. Therefore, a possible relay must first wake up the sink again by direct addressing it to indicate that it is acting as a relay. Due to this, this strategy requires additional WuPts and acknowledgements that are time and energy-consuming. The approach we have adopted, on the other hand, ensures that delay minimisation takes place by synchronously waking up the sensor nodes and the energy-efficient communication is achieved by means of no additional coordination packets.

Researchers extended in [24] the Collection Tree Protocol (CTP) [25], the de facto standard for data collection in WSN to work with nodes equipped with WuRx. CTP-WUR utilises WuRx to relay wake-up requests and reduce end-to-end data latency. By reducing the number of hops needed to relay data packets, the proposed approach improves the performance of using short-range WuRx scenarios. Each node in the network is assigned a unique WuRx address, a unique WuRx relay address, and a broadcast WUR address shared by all nodes in the network. In broadcast mode, each node in the sender’s neighbourhood will wake up and wait for receiving a data packet. On the contrary, in the unicast mode, the WuPts will be relayed without the need for waking up their main radio. Relay of WuPts is requested by means of a WuRx address containing the node’s unique address and an additional flag indicating that the WuPt needs to be forwarded to the receiver’s own parent. Compared to our work, the authors of this paper use the WuRx as a fully functional radio. They are able to receive and send WuPts with the WuRx, leaving the main radio in sleep mode. Even if the relay of the WuPt takes place without activating the main radio, energy is consumed for the forwarding of the WuPts.

In WHARP [26], authors propose a data forwarding strategy for green wireless networks that exploits the self-powered Wake-up Radio capabilities of the network nodes. The described strategy offers the possibility to send data to the destination by making decentralised and proactive decisions. The decision is made based on forecasting energy and expected traffic. To start communication, a WuPt is broad-casted and all recipients then decide whether they can become active and act as a relay. Sending a large number of coordination packets results in a lot of data traffic and an increased time of the sensor nodes in the active state. Since sending and receiving in wireless sensor networks is very energy consuming, this is to be avoided. As our approach determines which nodes are to be woken up or used as relays before communication begins, there is no unnecessary energy consumption due to unnecessary waking ups. In addition, our strategy does not generate unnecessary communication traffic and thus favours a time-efficient network.

A cross layer routing protocol called T-ROME was proposed in [27]. Authors introduce a set of parameters to optimise the relaying of data by dynamically choosing the most appropriate stopover nodes in case the sink is not reachable within one hop. The data communication in this strategy is based on forwarding of WuPts. This means that if the destination cannot be woken up directly, the relays forward the WuPt. If the destination is woken up, the source starts sending a data packet. Unlike our approach, where all routing information is stored at the Fog node, here, all sensor nodes have to determine the routes opportunistically on their own. This requires a large number of packets to be sent, which has a negative impact on the overall efficiency of the network. Since the relays have to receive and retransmit WuPts, another drawback in time and energy consumption occurs.

The work published in [28] is the Load Balancing Parent Selection (LoBaPS) protocol that supports opportunistic parent selection. In this strategy, the WuPt contains information about the node’s own rank (in a layered topology) together with a unique application ID (enumeration of WuPts). Each node in the communication range of the sender will receive this WuPt and compare the received rank with its own rank, and only wakes up its main radio if the former is higher than the own rank. In this case, each sensor node receives the DataPt sent by the source. However, only the sensor node that has the shortest CSMA (Carrier Sense Multiple Access) backoff forwards the WuPt followed by the data packet. The significant disadvantage of this strategy is the amount of energy wasted in listening mode when all the feasible successors wake up their main radio. Since only one sensor node per rank is included in the data forwarding process, but all sensor nodes in this rank receive the WuPt as well as the data packet, this leads to significant energy consumption. In addition, the most reliable route is chosen every transmission round. This leads to an uneven distribution of energy consumption and, therefore, most used nodes acting as relays can prematurely run out of power. In contrast, the approach we have developed has the advantage that sensor nodes, which are actually necessary for data forwarding, are specifically awakened. As the entire communication route is already known, it enables the Fog node to realise a time-efficient and energy-saving wake-up and data transmission.

Authors in [29] propose a strategy for multi-hop wake-up relay for CTP-WuR in which the Collection Tree Protocol is modified to work with Wake-up Radios. The feature of this approach is that the intermediate nodes are not woken up; they only forward the wake-up signal. If a node wants to wake up its parents two wake-up signals need to be transmitted. The first signal contains the address of the node via which the wake-up signal must be forwarded by means of the WuRx. The second signal contains the address of the destination node that the message should ultimately reach. If the address contained in the second signal does not match with the address of a node, the node sends its own pair of WuPt signals to act as a relay. The disadvantage of this strategy is that multiple WuPts have to be sent to wake up a single sensor node. This leads to unnecessary sending procedures, which are energy and time inefficient. Compared to our work, it is not necessary to send WuPt several times. With our addressing mechanism, we are able to wake up individual sensor nodes in an energy-efficient manner. On the one hand, this means that fewer packets are sent, which leads to reduce the risk of collisions. On the other hand, our approach offers a time and energy saving advantage by sending the WuPt once.

The presented protocol, called G-WHARP [30], is a wake-up radio-based forwarding strategy for wireless networks equipped with energy harvesting capabilities aiming to minimise energy consumption and end-to-end latency. Wake-up semantic addressing (energy-related aspects based on a Markov Decision Process (MDP)) is used to avoid waking up devices with no forwarding availability. In this strategy, the initiator node sends a broadcast WuPt that wakes up all sensor nodes with the corresponding semantic. The sensor node reacts first to the received WuPt by sending an acknowledgement that it receives the data. However, this approach of waking up nodes results in considerable energy consumption that is not necessary. The disadvantage is based on the fact that sensor nodes that are not required for data transmission are unnecessarily awakened for transmitting acknowledgements. This is because sensor nodes that do not act as target nodes also send an acknowledgement in response to the reception of the WuPt and then only switch back to sleep mode after not receiving a data packet. Even if the WuPt is determined in advance by the MDP, this leads to unnecessary energy consumption. Here, our strategy with a predetermined route and activation of the relays by multicast WuPt offers a more optimal solution in aspects of energy and latency.

## 4. Proposed Approach

In our proposed system, there are two types of nodes: powerful nodes (in aspects of energy and transmission power) that act as the sink or Fog Nodes and energy-critical cluster nodes that act as cluster members. Each Fog Node is main powered and is able to send messages directly to every node in its cluster. Whereas, battery powered cluster members use very low transmitting power. For this reason, when a given cluster member node A is placed faraway from the Fog Node, intermediate nodes are needed to relay the data from that node A to its corresponding Fog node. There is hence an *asymmetric link* between each Fog Node and its cluster members.

The sensor nodes in each cluster are equipped with a WuRx and are only activated when required by a special RF signal (WuPt). If these sensor nodes are not activated, the entire sensor node remains in sleeping mode and thus does not consume any energy. As already mentioned, data communication within a wireless sensor network is a very energy-consuming process, which is why the individual sensor nodes use a reduced transmission power around −34 dBm. This ensures that these energy-critical sensors send data in an energy-efficient way.

**Figure 2:**
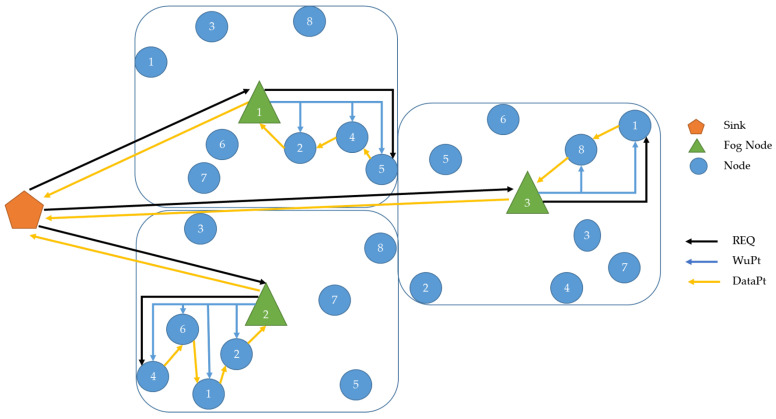
Scenario of asymmetric communication using multicast WuPt transmission using 1, 2, or 3 cluster Nodes as relay.

To return to the description of an individual cluster, as shown in Figure 2, we go into the formation in more detail. It should be noted that each cluster is assigned to a single Fog Node, to which eight sensor nodes are assigned. The Fog Node is the only network participant that enables communication between the sink and the individual sensor nodes of a cluster. However, data communication within a cluster is possible via multi-hop. This means that the individual sensor nodes can communicate with each other and send data packets to the Fog Node using cluster nodes as relays.

Focusing on an energy-efficient and delay-minimising communication strategy using WuRx, we have developed a novel energy- and time-efficient strategy. Since, in a WuRx-based WSN, the sensor nodes are in sleeping mode, it is necessary to wake up these nodes in order to use them for data transmission. The most trivial way is that each node sends a wake up message to the next node that will be used as the relay until reaching the destination. However, such a strategy is too time consuming because, in each step, we need to wait for the intermediate node to wake up and become ready to transmit the packet. In health care scenarios, such as in an elderly or Alzheimer’s patient’s household, the supervision of the patient is needed to have very short reaction time when a patient faces health problems. Therefore, even a few additional milliseconds make the difference in the health status of the patient. Thus, the said strategy is inconvenient for time-restricted application.

Our routing strategy aims to overcome this limit by allowing the Fog Node to wake up multiple sensor nodes simultaneously. This ensures a timely waking up of the nodes that will act as relays from the source to the Fog Node. To enable these multiple wake-ups, the Fog Node has a list of the addresses of all associated cluster members. To know which are the relay nodes that will be woken up, the Fog Node uses source routing. It maintains in its routing table the list of nodes that will participate in the routing process.

### 4.1. Node Description

**Figure 3:**
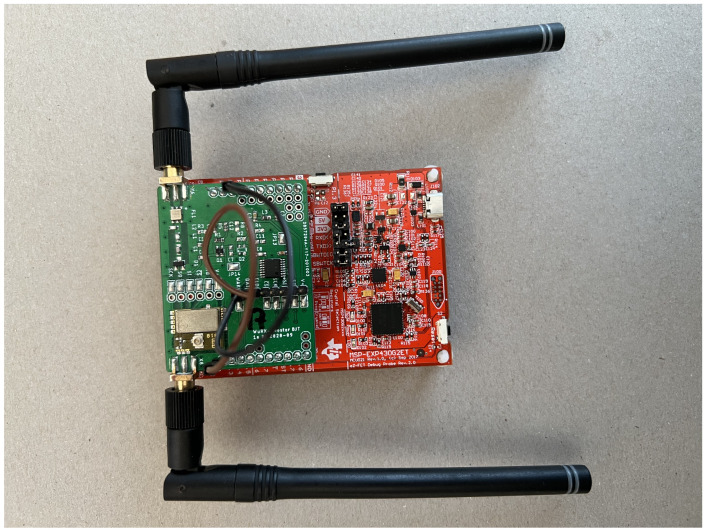
Wake-up Receiver equipped sensor node used in this work.

The wireless nodes used in this work are based on commercial off-the-shelf (COTS) components that are usually cheaper in setup, maintenance, expansion, and development. In order to save energy, the used WuRx equipped with a 3.0 V battery uses passive components for continuous listening to the radio channel. Figure 3 shows a photo of the implemented sensor node. The MCU utilised on the boards is a 16-bit MSP430G2553 [31] microcontroller operating at 8 MHz and manufactured by Texas Instruments. The MSP430 can enter multiple low-power modes; when in Low-Power Mode 3 it consumes 2.55 µW. The communication radio is a SPIRIT1 [32] radio module from STMicroelectronics. It has a current consumption of 21 mA when transmitting at +12 dBm output power at 868 MHz and about 9 mA when receiving with an approximated sensitivity of −118 dBm. The LF WuRx chip AS3933 is a low power, 3-channel ASK receiver with a current consumption of around 3 µA in listening mode. The AS3933 [33] is designed for carrier frequencies of 15 kHz to 150 kHz using On-Off-Keying (OOK) modulation. The modulated OOK signal of 18.7 kHz is converted to the carrier signal of 868 MHz which, when received by a sensor node, is converted back to the kHz band by the passive envelope detector. The AS3933 correlates the incoming signal with the node-specific address and generates an interrupt if both addresses match.

### 4.2. Wake-Up Packet Addressing

**Figure 4:**
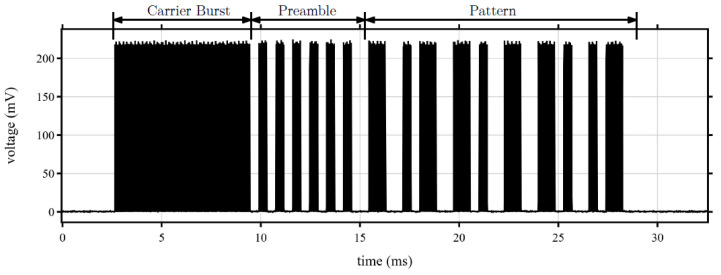
Manchester-coded wake-up signal consisting of carrier burst, preamble, and address pattern [34].

In our approach, wake-up signals consisting of carrier burst, preamble, and address pattern are used and shown in Figure 4. We assign WuRx addresses made up of a pattern of 16 bits to the sensor nodes equipped with a WuRx. The first 8 bits of the address represent the assignment to a cluster. The remaining 8 bits represent the unique address of the individual sensor nodes within the cluster. This results in a two-part address for multicasting. In order to use the WuRx address required for multicast wake up, the Fog Node accesses a table in which the multicast WuPt addresses are stored. It should be noted that 8 sensor nodes with WuRx are assigned to each cluster. This means that for each of the sensor nodes, there is a unique assignment of the rear 8 bits of the WuRx address.

**Figure 5:**
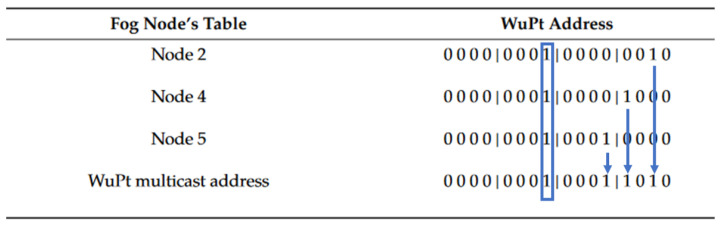
Example of forming the multicast wake-up pattern.

To illustrate this, based on the scenario outlined in Figure 2, in the first cluster using two cluster nodes as relay, Figure 5 is given. It can be seen that the Fog Node needs to wake up sensor nodes 2 and 4 for the data communication of cluster node 5, as these nodes must act as relays. This results in the address to be used for the WuPt for the Fog Node. According to the previous description, the bit corresponding to cluster 1, i.e., bit 8, is now set in the multicast WuPt and highlited in the Figure with the blue outline. To wake up the individual sensor nodes 2, 4, and 5, bits 1, 3, and 4 are set to 1 in the rear half of the address pattern according to the assignment as illustrated with the blue arrows. This will generate the multicast address shown in the graphic.

If a sensor node equipped with a WuRx receives the described WuPt, a bit-by-bit comparison of the addresses is started. Therefore, if the WuPt pattern for the cluster assignment bit (and the unique bit in the rear part if the address match those of the sensor node), an interrupt is generated and the entire sensor node is activated. If there is no positive bit matching, the sensor node goes into sleep mode.

Our proposed smart addressing scheme offers the possibility to activate nodes in the routing path without increasing delays. Thus, we achieve a very prompt and energy-efficient sensor awakening.

### 4.3. Routing Process Description

**Figure 6:**
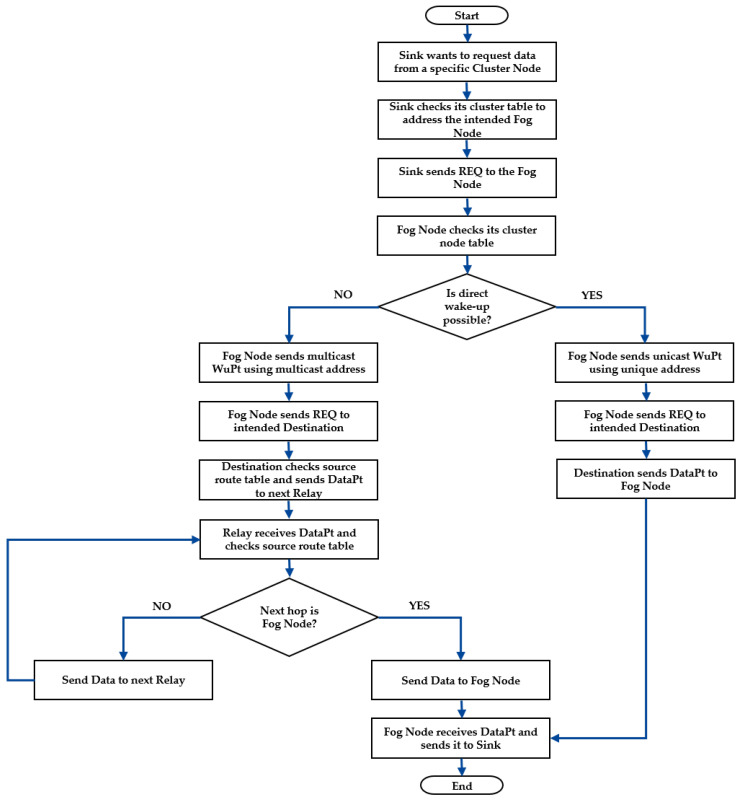
Communication using the proposed wake-up approach.

The key idea of the proposed method is that the sink performs transmission scheduling while the Fog Node uses a multicast strategy to wake up nodes for asymmetric data communication. The process is depicted in the Algorithms 1 and 2 and summarised as a flow diagram shown in Figure 6.

1. The SINK decides the routing and transmission timing of each sensor node. When the SINK tries to collect sensor data from the Destination Node DST, it checks its routing table to find out which Fog Node FN is in charge for the DST. Then the SINK transmits an REQ to the responsible FN.
2. The FN is always on and main powered. It receives the request REQ from the SINK and checks its routing table to specify the nodes that will act as Relays REL. The wake-up messsage WuPt is sent via multicast to wake up the DST and its corresponding RELs. The FN remains in receiving mode.
3. The DST receives the WuPt and starts data transmission to the REL. Each node that finalises its transmission returns to the sleeping mode.
4. The FN receives the data and forwards the packet to the SINK. The FN goes to receive mode.
5. The SINK receives the data.

**Algorithm 1:** WuPt transmission
1:
**procedure** waking up nodes using multicast WuPt (SINK, FN, REL, DST)
2:    **if** *Sink requests data from DST* **then**3:        SINK checks cluster table for addressing responsible FN sending REQ to FN4:        **if** *FN receives REQ* **then**5:           check cluster member table6:           **if** *DST is in direct range* **then**7:               send unicast WuPt8:           **else** *send multicast WuPt for DST and REL*


**Algorithm 2:** Data transmission
1:
**procedure** Data forwarding after multicast WuPt (SINK, FN, REL, DST)
2:    **if** *WuPt is received* **then**3:        bit-wise check of WuPt address4:        **if** *cluster-bit and ID-bit = = own cluster-bit and ID-bit* **then**5:           wake-up sensor node and switch to receive mode6:           **if** *receiving request from FN* **then**7:               check source route table8:               **if** *next hop != FN* **then**9:                   check source route table and send DataPt to REL10:               **else** *send Data to FN and go to sleep mode*11:               **end procedure**.12:           **else** *receive DataPt* and check source route table13:               **if** *next hop != FN* **then**14:                   check source route table and send DataPt to REL15:               **else** *send Data to FN and go to sleep mode*16:        **else** *go to sleep mode*


## 5. Experimental Setup and Measurement

The measurements were carried out indoor in the laboratory at the Leipzig University of Applied Sciences. The cluster size is based on 1 Fog Node and 8 sensor nodes. All nodes and Fog Node are static and distributed over a 30 m × 30 m plane. The sink is located outside of the cluster. In order to get more comparative results, we varied the number of relaying nodes from 1 to 3. We compared the different strategies and evaluated the consumed energy and the end-to-end delay.

### 5.1. Description of the Proposed Approach and the Compared Works

**Figure 7:**
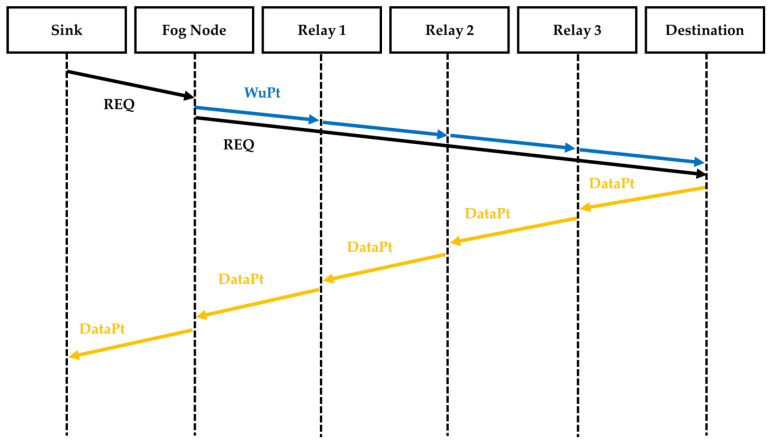
Sequence diagram of the proposed approach CWM using multicast WuPt transmission.

**Figure 8:**
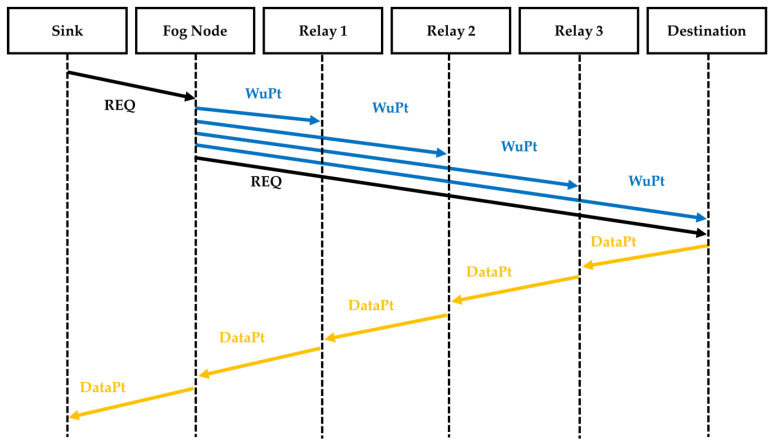
Sequence diagram of the SBS approach.

We compare our proposed approach depicted in Figure 7 to the technique described in Figure 8. In this approach, called Step-by-Step (SBS), after receiving the REQ from the sink, the Fog Node sequentially wakes up each sensor node used as a relay and the destination last. Following the forwarding of the REQ from the Fog Node to the destination node, the destination starts sending data using the woken up relays. After sending the data, the sensor nodes all go back into sleep mode.

**Figure 9:**
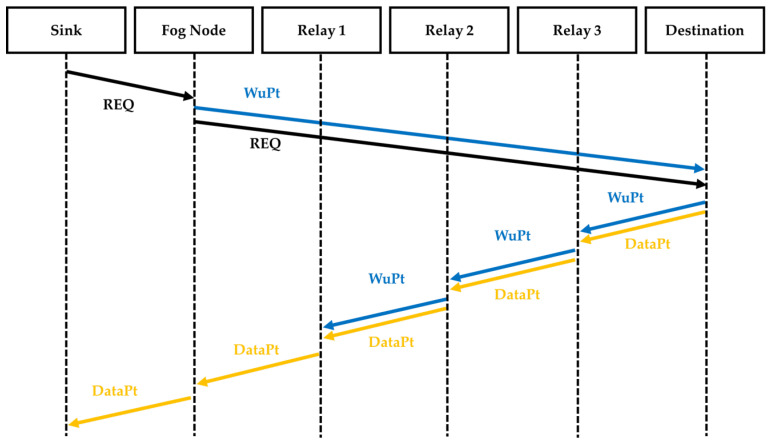
Sequence diagram of NTN approach.

The second strategy that we used in the comparison, called Node-to-Node (NTN), is described in Figure 9. Here, after receiving the REQ from the sink, the Fog Node sends a WuPt to the destination followed by the forwarding of the REQ. Then, the destination node sends a WuPt to the next relay followed by a data packet. After waking up and receiving the data packet, the relay sensor node sends another WuPt and transmits the data packet to the next relay. The next relay wakes up and receives the data packet, which it sends on. After transmitting the data, all the sensor nodes return to sleep mode.

### 5.2. Experimental Setup

In the following, the parameters used during the experimental measurements are listed in Table 1. At this point, we would like to give a note on the hardware used. For Fog Node, we have used Raspberry Pi 4 [35]. As already mentioned, off-the-shelf components were used to build the WuRx sensor node, which consist of the AS3933 as the WuRx, the SPIRIT1 as the transceiver, and the MSP430 as the microcontroller.

### 5.3. Performance Evaluation

**Figure 10:**
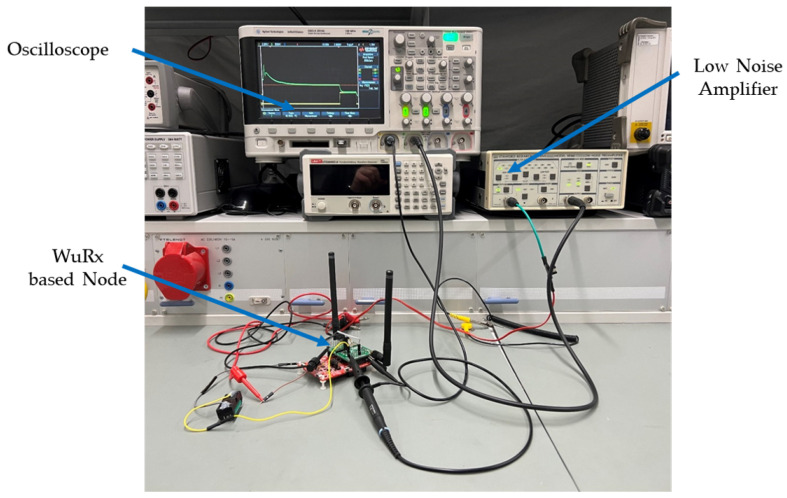
Set up used for measurements.

For experimental acquisition of the measurement data and the subsequent evaluation of the results, the energy consumption in ampereseconds and the time in active mode in milliseconds of all sensor nodes was measured using an oscilloscope. The technical setting for the analysis as shown in Figure 10 also includes a shunt resistor of 1 Ω and a low noise amplifier to amplify the signal by a factor of 100. In order to better understand the results of the energy consumption measurement of each sensor node, a measurement of the active times of each individual node was carried out. The measurement results represent the energy consumption and time each node sends, waits or receives an REQ, WuPt, or DataPt. The measurement carried out using the described measurement setup and the resulting outcomes follow subsequently.

**Figure 11:**
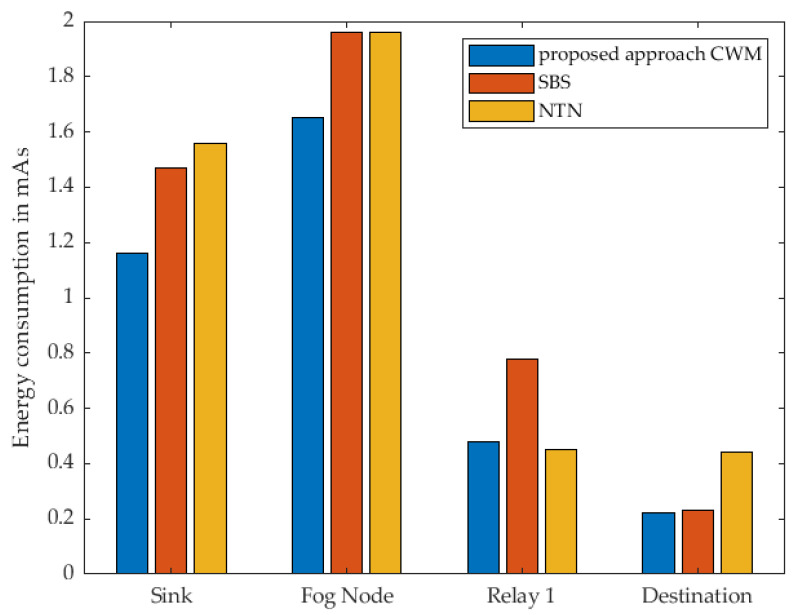
Measured energy consumption of every single node using 1 Relay.

In Figure 11, the energy consumption of every single node within the communication route when using 1 cluster node as relay at the different strategies is shown. The results show, that when using our proposed approach, almost each individual sensor node consumes the least energy compared to the other two strategies. It is noticeable that the Destination Node consumes almost the same amount of energy in our approach and the strategy named SBS. Based on the previous description of the different strategies by means of the sequence diagram, it is evident that this relay is identical in terms of the activities and also in terms of the time in active mode. Both nodes receive a WuPt followed by transmitting a DataPt to the Cluster Node acting as relay. Since the actions are the same, they should consume the same amount of energy and take the same amount of time. This minimal difference can be attributed to a variety of reasons. Indoors, wireless sensors are generally more difficult to use due to signal fading and reflections. It should also be noted that these are real measurements using prototypes that are not entirely identical to each other, which leads to minimal deviations in the collected measurement data. Since this minimal difference cannot be eliminated, and the variation is that minimal and has no significant influence on the overall assessment of the different scenarios, this deviation can be regarded as not significant. Even if this anomaly is noticeable here in this scenario with one relay, it does not have much effect in the following measurements with several relays.

**Figure 12:**
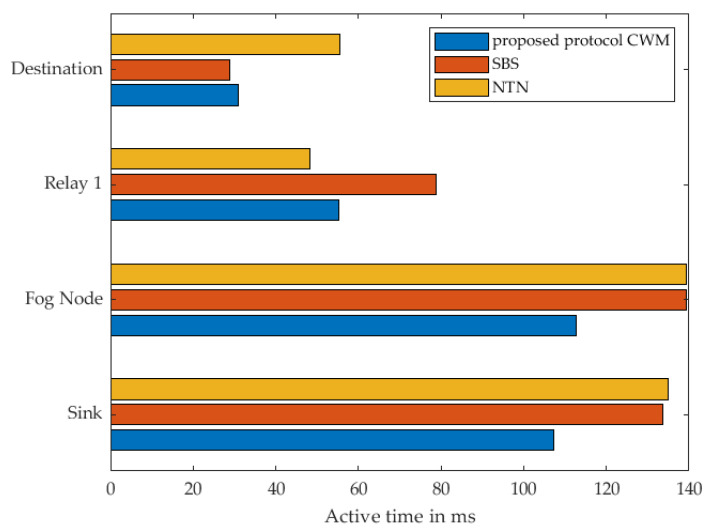
Measured time of every single node in active mode using 1 Relay.

The total active-mode time for each individual sensor node is shown in Figure 12. When considering our approach and SBS, it is noticeable that according to the sequence diagrams in Figure 7 and Figure 8, the destination node performs the same actions. As already stated, differences between the individual nodes can also be determined in these results. The specific results can be found in Table 2 and Table 3.

**Figure 13:**
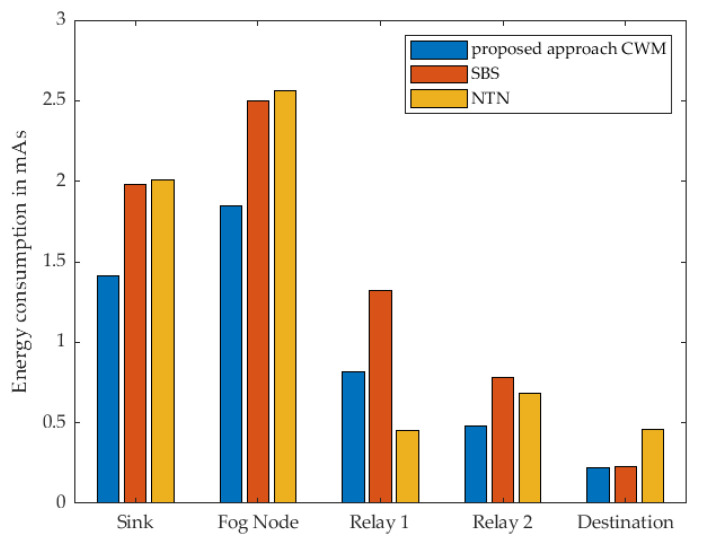
Measured energy consumption of every single node using 2 Relays.

**Figure 14:**
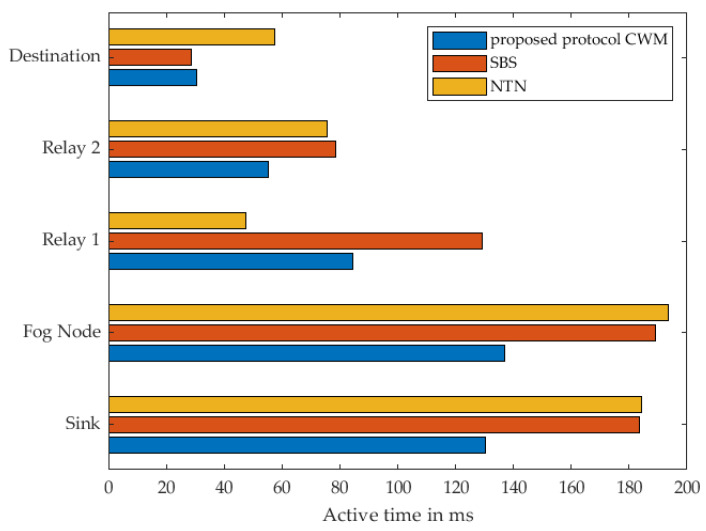
Measured time of every single node in active mode using 2 Relays.

The measurement results for the different strategies with two relay nodes are shown in Figure 13. It can be seen that the proposed approach has the best efficiency in terms of energy consumption in almost all sensor nodes. The exception is Relay 1. The reason for this is that Relay 1 is woken up at the same time as Relay 2 and the destination in the sense of simultaneous multicast wake-up. This means that Relay 1 remains in the receive mode for a certain time until it receives the DataPt. The behaviour is similar with SBS. As far as Relay 1 is concerned, NTN is the strategy that is very energy efficient. This is due to the fact that with the NTN strategy the sensor nodes wake each other up one after the other. Thus, the relay is only woken up for on-demand and never remains in receive mode unnecessarily. Figure 14 shows the according time in active mode. The specific results can be found in Table 4 and Table 5.

**Figure 15:**
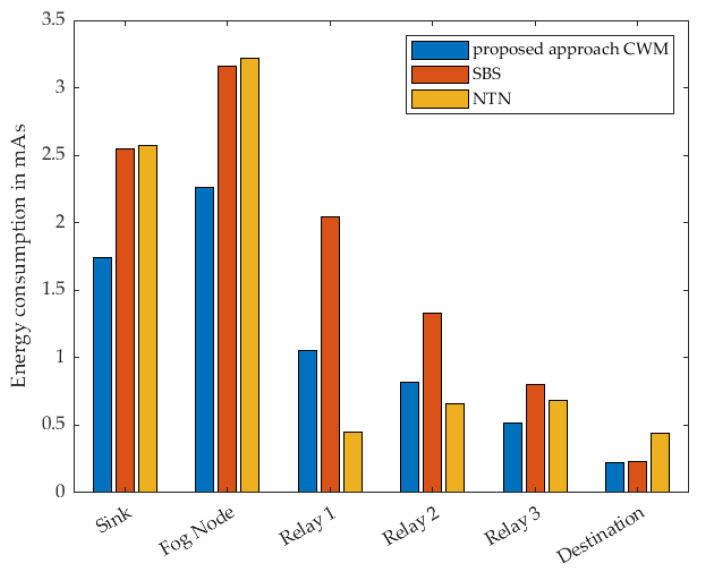
Measured energy consumption of every single node using 3 Relays.

**Figure 16:**
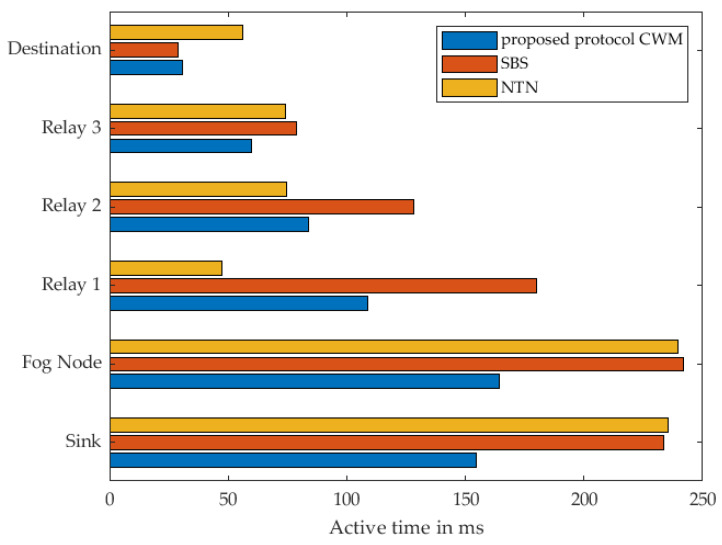
Measured time of every single node in active mode using 3 Relays.

The maximum number of relays considered within this study was three cluster nodes. The corresponding result of this measurement is shown in Figure 15. As with the previous results, a similar behaviour can be seen with regard to the consumed energy. In our proposed approach it is particularly notable that Relay 1, i.e., the node that sends the DataPt to the Fog Node last, always consumes the most energy. As described earlier, this is due to the time the node waits for a DataPt in receive mode. This is where NTN’s strategy shows its strength, that relays are only woken up when they are needed. The SBS approach clearly shows how long waiting in receive mode affects energy consumption. The same behavior as in previous measurements can also be seen in the course of the active states of the individual nodes over time presented in Figure 16. The specific results can be found in Table 6 and Table 7.

**Figure 17:**
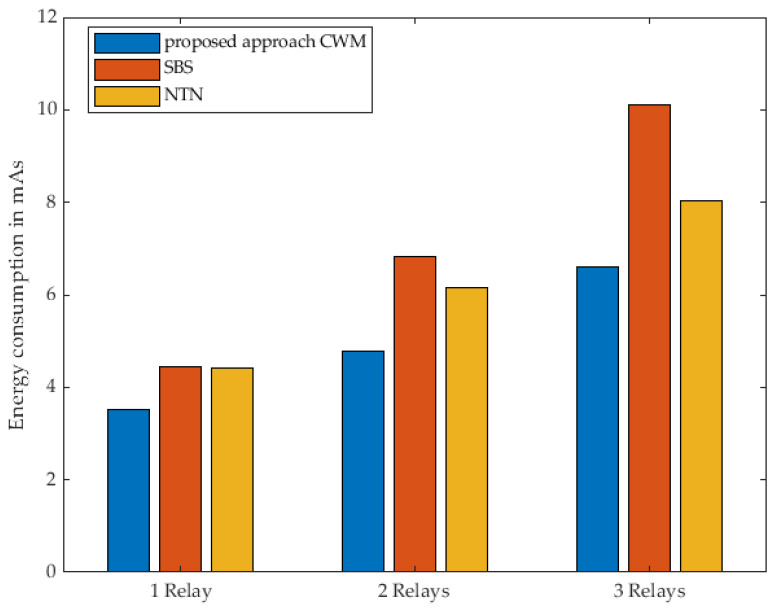
Nodes of energy consumption related to the number of relay nodes comparing the 3 strategies.

Figure 17 and the corresponding Table 8 present, respectively, the overall consumed energy with respect to number of cluster nodes used as relays. The different strategies based on the number of relays used for data transmission were considered to understand how the energy consumption changes when the communication path is extended.

The results show that the benefit regarding the energy consumption is given since our approach consumes less energy than the other compared protocols in all network sizes. The reason for this is that although the sensor nodes used for data transmission are all woken up at the same time, the Fog Node has only to send one single WuPt. The unique sending and simultaneous waking up of the cluster nodes leads to all of the used nodes being in active mode and ready to react immediately.

Compared to SBS, which enables sequential transmission of WuPts, the longer activity state in the active mode of the relays, as well as the multiple transmission of WuPts, results in increased energy consumption. The approach followed in NTN, in which the destination node is woken up directly, also presents increased energy consumption. This is due to the fact that each of the relays has to send WuPt in addition to the actual data packet. This increased sending of packets leads to a non-negligible energy consumption.

Regarding all three strategies, we can clearly see the superiority of our approach in terms of energy-efficiency. Regardless of the number of relays, our approach delivers the best result in all measurements.

**Figure 18:**
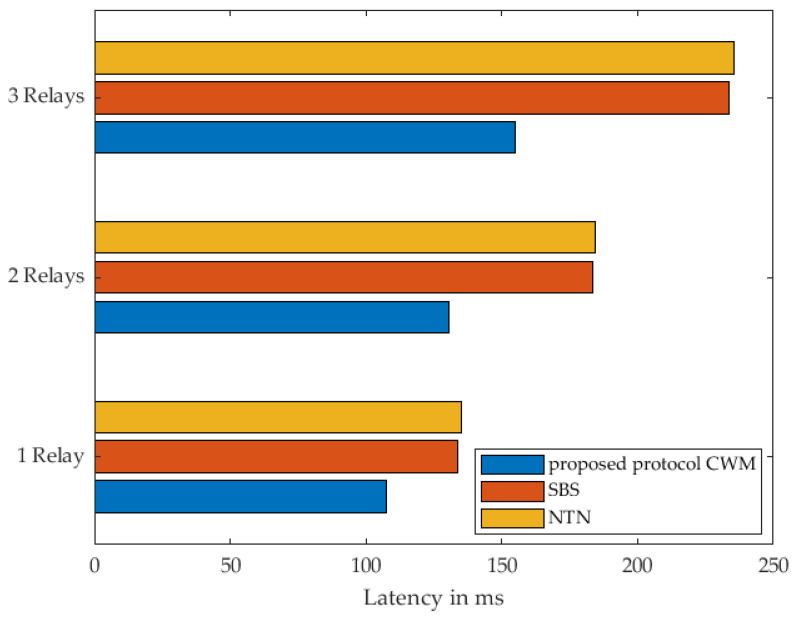
Routing latency with respect to the number of relay nodes comparing the 3 strategies.

Figure 18 depicts the performance of the different strategies in terms of packet transmission delay with respect to the different number of relays participating in the transmission route. The specific results can be found in Table 9. As shown in this figure, our approach drastically outperforms both of the other strategies in shorter communication delay, and the more the number of relay nodes increases, the more the out-performance of our proposed approach becomes clearer. This is due to the fact that the simultaneous wake-up of the sensor nodes used for data transmission takes place at the same time, which saves time and leads to direct data transmission as soon as the destination node is awakened. Considering the results of SBS strategy, which allows sequential sending of the WuPts, an increased time demand is required compared to our strategy. This is based on sending the WuPts one after the other. Depending on the number of relays, a corresponding number of WuPts are required to enable data transmission. This frequent sending, therefore, leads to an increased delay. For the NTN strategy, the destination node is woken up directly, and each of the relays must send WuPt in addition to the actual data packet. This increased sending of packets leads to an increased delay in data communication.

The aforementioned results show the superiority of our approach that offers the best trade-off between time-efficiency and energy optimisation. Regardless of the number of relays, our approach delivers the best result in all measurements. However, the greater the number of relays increases, the more the clearer and more significant the advantages of our approach become. Particularly in time-critical applications such as medical, monitoring, and alarm situations in the home area, the advantages of our approach become apparent.

In the following, we study the lifetime of our CWM protocol as well as the two other strategies. The lifetime in our scenario is the time needed until the first node in the network is out of energy. The battery-powered cluster nodes are powered by a standard CR2032 coin cell with a capacity of 240 mAh at 3 V. With an occurrence of events every 3 min; the lifetime for our proposed approach in the scenario with only 1 relay is 10.27 years, for SBS 6.32 years, and for NTN 10.96 years. Increasing the number of relays to two results in a lifetime of 6.01 years for our proposed approach, whereas SBS results in 3.74 years and NTN in 7.25 years. With the maximum number of relays considered in this work, three relays, the lifetime was 4.70 years with our proposed approach, 2.42 years for SBS, and 7.25 years for NTN.

Considering the time required for data communication with the individual strategies, our proposed approach outperforms the other strategies by offering the best trade-off between energy efficiency and delay minimisation. The delay minimisation of our strategy was 19.75% for SBS and 20.55% for NTN. Even when using two relays, our approach has an advantage of 28.92% over SBS and 29.23% over NTN. With three relays, our approach had a delay minimisation of 33.7% compared to SBS and 34.25% compared to NTN.

## 6. Conclusions

In this paper, we have proposed an energy-aware and delay-minimising routing protocol for heterogeneous Wake-up Receiver-based WSNs. Due to the different participants in a cluster, sink and Fog Node have more capabilities in terms of transmission power and energy budget than the individual sensor nodes. Each sensor node is equipped with a WuRx. When a sink wants to get data from a given sensor, it sends the request to its corresponding Fog Node. This latter wakes up this destination node as well as the intermediate relays using a novel multicast addressing scheme.

Experimental results have shown that our proposed approach gives better results than the two strategies we compared it with. The simultaneous waking up of the destination and the relay acting nodes thus offers the possibility of shortening delays and saving energy, since multicast eliminates the need to send several WuPts.

In the future work, we will further increase the communication opportunities of the network participants. It is necessary to expand the strategy and the addressing mechanism in order to achieve scalability. Even considering an indoor scenario, the expansion of a cluster is a necessary task. We aim also to enable inter-cluster communications via Fog Nodes. This has the advantage that if direct communication between a Fog Node and the sink is not possible, communication can take place via another intermediate Fog Nodes. Furthermore, inter-cluster communication can be used to address clusters that cannot be reached directly from the sink in large-scaled networks. We plan also in our future work to take into account the case of Fog Node failure and propose a healing strategy that enables the simple nodes to carry out inter-cluster communication. These nodes should then either be able to wake up nodes of a neighbouring cluster or, if possible, directly address the neighbouring Fog Node.

## Figures and Tables

**Table 1:** Parameter values used in this experimental measurement.

Parameter	Value
Strategies	proposed approach CWM, SBS, NTN
Hardware	AS3933 (WuRx), SPIRIT1 (Transceiver), MSP430 (MCU), Raspberry Pi 4
WuPt	Mancherst Bits (8 Carrier Burst, 6.5 Preamble, 16 Address)
DataPkt	size 107 Byte (8 Byte Preamble, 4 Byte Sync, 1 Byte Length, 1 Byte Address, 92 Byte Payload, 1 Byte CRC)
REQ	size 18 Byte (8 Byte Preamble, 4 Byte Sync, 1 Byte Length, 1 Byte Address, 3 Byte Payload, 1 Byte CRC)
Frequency band	868.0 MHz
Bit-rate	AS3933 (1.16 kbit/s), Spirit (38.4 kbit/s), MSP430 (8 Mbit/s)
Modulation	AS3933 (OOK), SPIRIT1 (FSK)
Area	Indoor 30 m × 30 m
Transmission power Sink and Fog Node	WuPt and DataPt (+12 dBm)
Transmission power Cluster Nodes	WuPt (−6 dBm) and DataPt (−34 dBm)

**Table 2:** Single nodes energy consumption using 1 Relay.

Relay	Proposed Approach CWM mAs	SBS mAs	NTN mAs
Sink	1.16	1.47	1.56
Fog Node	1.65	1.96	1.96
Relay 1	0.48	0.78	0.45
Destination	0.22	0.23	0.44

**Table 3:** Single nodes time in active mode using 1 Relay.

Relay	Proposed Approach CWM ms	SBS ms	NTN ms
Sink	107.30	133.70	135.05
Fog Node	112.58	139.39	139.46
Relay 1	55.26	78.69	48.14
Destination	30.85	28.86	55.62

**Table 4:** Nodes energy consumption using 2 Relays.

Relay	Proposed Approach CWM mAs	SBS mAs	NTN mAs
Sink	1.41	1.98	2.01
Fog Node	1.85	2.50	2.56
Relay 1	0.82	1.32	0.45
Relay 2	0.48	0.78	0.68
Destination	0.22	0.23	0.46

**Table 5:** Nodes time in active mode using 2 Relays.

Relay	Proposed Approach CWM ms	SBS ms	NTN ms
Sink	130.46	183.55	184.34
Fog Node	137.06	189.04	193.35
Relay 1	84.60	129.27	47.52
Relay 2	55.12	78.58	75.77
Destination	30.52	28.81	57.66

**Table 6:** Single nodes energy consumption using 3 Relays.

Relay	Proposed Approach CWM mAs	SBS mAs	NTN mAs
Sink	1.74	2.55	2.57
Fog Node	2.26	3.16	3.22
Relay 1	1.05	2.04	0.45
Relay 2	0.82	1.33	0.66
Relay 3	0.52	0.80	0.68
Destination	0.22	0.23	0.44

**Table 7:** Single nodes time in active mode using 3 Relays.

Relay	Proposed Approach CWM ms	SBS ms	NTN ms
Sink	154.80	233.50	235.43
Fog Node	164.41	242.13	239.38
Relay 1	108.69	180.12	47.41
Relay 2	83.81	128.30	74.37
Relay 3	59.85	78.69	74.37
Destination	30.62	28.77	56.04

**Table 8:** Nodes of energy consumption related to the number of relay nodes comparing the 3 strategies.

Relay	Proposed Approach CWM mAs	SBS mAs	NTN mAs
1 Relay	3.51	4.45	4.42
2 Relays	4.77	6.82	6.15
3 Relays	6.59	10.10	8.03

**Table 9:** Time consumption related to the number of relay nodes comparing the 3 strategies.

Relay	Proposed Approach CWM ms	SBS ms	NTN ms
1 Relay	107.30	133.70	135.05
2 Relays	130.46	183.55	184.34
3 Relays	154.80	233.50	235.43
